# PARP1-targeted alpha therapy enhances target expression

**DOI:** 10.1186/s13550-025-01256-0

**Published:** 2025-06-01

**Authors:** Hasan Babazada, Paul Martorano, Hsiaoju Lee, Shuyao Geng, Vandana Batra, John M. Maris, Daniel A. Pryma, Sarah B. Gitto, Michael D. Farwell

**Affiliations:** 1 Perelman School of Medicine at the University of Pennsylvania, 3620 Hamilton Walk, John Morgan Bldg., Philadelphia, PA 19104 USA; 2 Children’s Hospital of Philadelphia, Philadelphia, PA USA; 3 Abramson Cancer Center at the University of Pennsylvania, Philadelphia, PA USA

**Keywords:** Alpha particle therapy, PARP1, PET/CT, Neuroblastoma, DNA damage, Astatine-211

## Abstract

**Supplementary Information:**

The online version contains supplementary material available at 10.1186/s13550-025-01256-0.

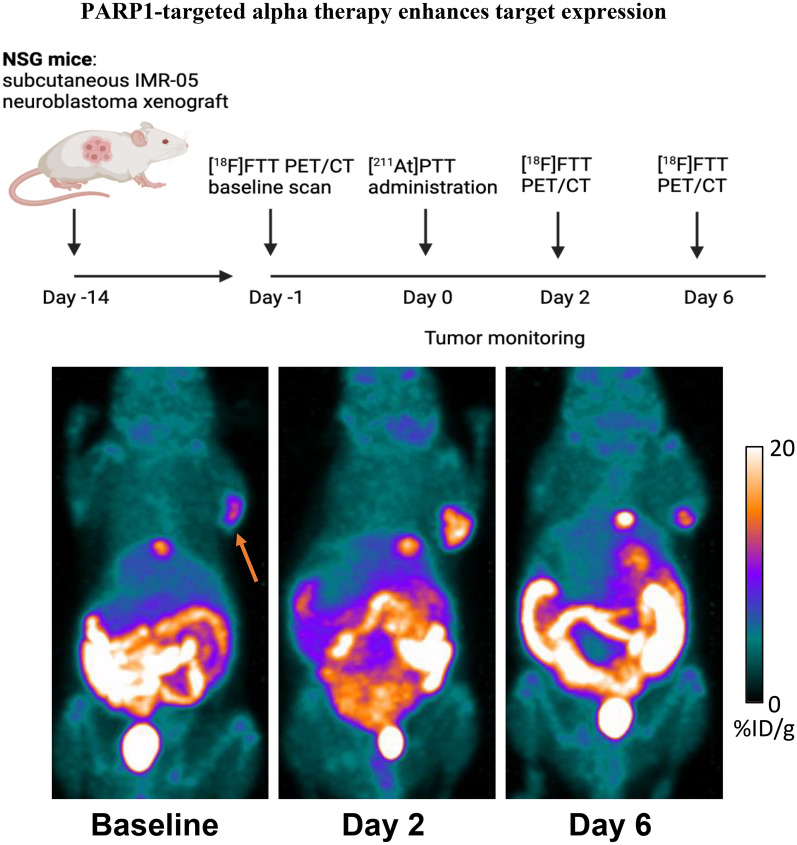

## Introduction

High-risk neuroblastoma, the most common extracranial solid tumor in children, is associated with a poor prognosis, with survival rates below 50% despite multimodal therapies [1]. Targeted radiopharmaceutical therapy, particularly alpha-particle emitters such as ^211^At, has emerged as a promising approach due to its ability to deliver precise, high-energy radiation that induces irreparable DNA damage [2–8]. However, sublethal doses of radiation may leave damaged cells susceptible to relapse or resistance. To address this challenge, we hypothesized that targeting poly(ADP-ribose) polymerase 1 (PARP1), a key enzyme in DNA damage repair, could increase its expression in sublethally damaged cells. This upregulation could enhance the efficacy of fractionated therapy by rendering tumor cells more vulnerable to subsequent DNA damage.

[^211^At]-parthanatine (PTT), a PARP1-targeted alpha-particle therapy, has demonstrated potent cytotoxicity in neuroblastoma models [3, 4, 9]. To monitor target engagement and predict therapeutic response, we paired [^211^At]PTT with [^18^F]-fluorthanatrace (FTT), a PARP1-specific PET imaging agent [10]. This study aimed to evaluate whether [^211^At]PTT increases PARP1 expression in neuroblastoma xenografts, as visualized by [^1^^8^F]FTT PET/CT imaging, and to assess DNA damage (via γH2AX) and the potential for fractionated therapy.

## Materials and methods

### Radiochemistry

[^18^F]FTT was produced at the University of Pennsylvania Cyclotron Facility, following the method previously described by Zhou et al. [11]. The specific activity of [^18^F]FTT ranged from 78.8 to 102.3 GBq/µmol, with a radiochemical purity of ≥ 99.4% (Supplementary Fig. S1). [^211^At]PTT was synthesized via electrophilic aromatic destannylation of a tin precursor, as adapted from the method described by Reilly et al. [9]. The specific activity of [^211^At]PTT was 16.6 GBq/nmol, with a radiochemical purity of 100% (Supplementary Fig. S2).

### Cell culture

IMR-05 human neuroblastoma cells were cultured at 37 °C with 5% CO_2_ in RPMI-1640 medium supplemented with 10% FBS and 1% non-essential amino acids. Cells were routinely tested for mycoplasma and confirmed mycoplasma-free before experiments.

### Cell treatment and RNA extraction

IMR-05 neuroblastoma cells were seeded in 6-well plates at 2 × 10^5^ cells/well in 2 mL medium. After 24 h, cells were treated with [^211^At]PTT at final concentrations of 3.7, 9.25, 18.5, and 37 kBq/mL. Controls received complete medium without [^211^At]PTT. Cells were harvested at 3, 6, 24, and 48 h, washed with PBS, and lysed with RLT buffer containing β-mercaptoethanol. RNA was extracted using the RNeasy Mini Kit, eluted in RNase-free water, and quantified using a NanoDrop 2000. RNA yields were recorded for analysis across time points and treatments.

### cDNA synthesis and quantitative real-time PCR (qPCR)

cDNA was synthesized from 1 µg RNA using the RevertAid First Strand cDNA Synthesis Kit (42 °C for 60 min, 70 °C for 5 min), then diluted 1:10 for qPCR. qPCR was performed on a QuantStudio 3 system using TaqMan Gene Expression Master Mix and assays for PARP1 and B2M (internal reference). Cycling conditions: 50 °C for 2 min, 95 °C for 20 s, followed by 40 cycles of 95 °C for 1 s and 60 °C for 20 s. PARP1 expression was calculated using the ΔΔCt method, normalized to B2M and referenced to untreated controls.

### Animal studies: [^18^F]FTT PET imaging and [^211^At]PTT therapy

NSG mice (NOD/SCID IL2Rγ − / −) were obtained from the University of Pennsylvania and maintained under Institutional Animal Care guidelines. IMR-05 tumor-bearing mice (n = 9) were established by injecting 1 million IMR-05 cells mixed with 50% Matrigel in RPMI-1940 into the flanks of 6- to 8-week-old females. Baseline microPET imaging was performed two weeks post-implantation, 60 min after IV injection of 7.4 MBq [^18^F]FTT. A single 370 kBq dose of [^211^At]PTT was administered IV to the treatment group (n = 6) 24 h later, with follow-up imaging at 2 and 6 days post-treatment. Three mice were sacrificed at each time point to dissect tumors at baseline, day 2, and day 6 for γ-H2AX and PARP1 analysis. Imaging used X-Cube micro-CT and β-Cube PET scanners (energy window: 435–587 keV), with images reconstructed using OSEM (20 iterations). Tumor activity was measured, decay-corrected, and expressed as %ID/g using MIM Software. Tumor volumes were calculated as V = 0.5 × L ×W ^2^.

### Immunofluorescence

Tumors were resected, snap-frozen, embedded in OCT, and sectioned at 10 µm for immunofluorescence. Slides were washed with PBS, fixed in 4% PFA for 10 min, permeabilized with 0.1% Triton X-100 on ice for 10 min, and blocked with 10% goat serum for 60 min or overnight. Sections were incubated with primary antibodies (PARP1, 1:1000; γ-H2AX, 1:5000) for 1 h, washed, and exposed to secondary antibodies (Alexa Fluor 555 and 488) for 1 h. Slides were mounted with ProLong Glass containing NucBlue (Hoechst 33344). Imaging was performed using a Zeiss Widefield microscope, and analysis was done with Fiji (ImageJ). Background subtraction was applied, and Hoechst 33344 was used to identify nuclei. The total fluorescence intensity in each field of view analyzed at 20 × magnification was then normalized to the number of Hoechst 33344-positive nuclei to account for variations in cell density.

### Statistical analysis

Results are presented as mean ± standard deviation (SD) unless otherwise stated. Statistical analysis was performed using two-way ANOVA with Tukey–Kramer multiple comparisons test for unequal sample sizes (GraphPad Prism 10).

## Results

**Fig. 1:**
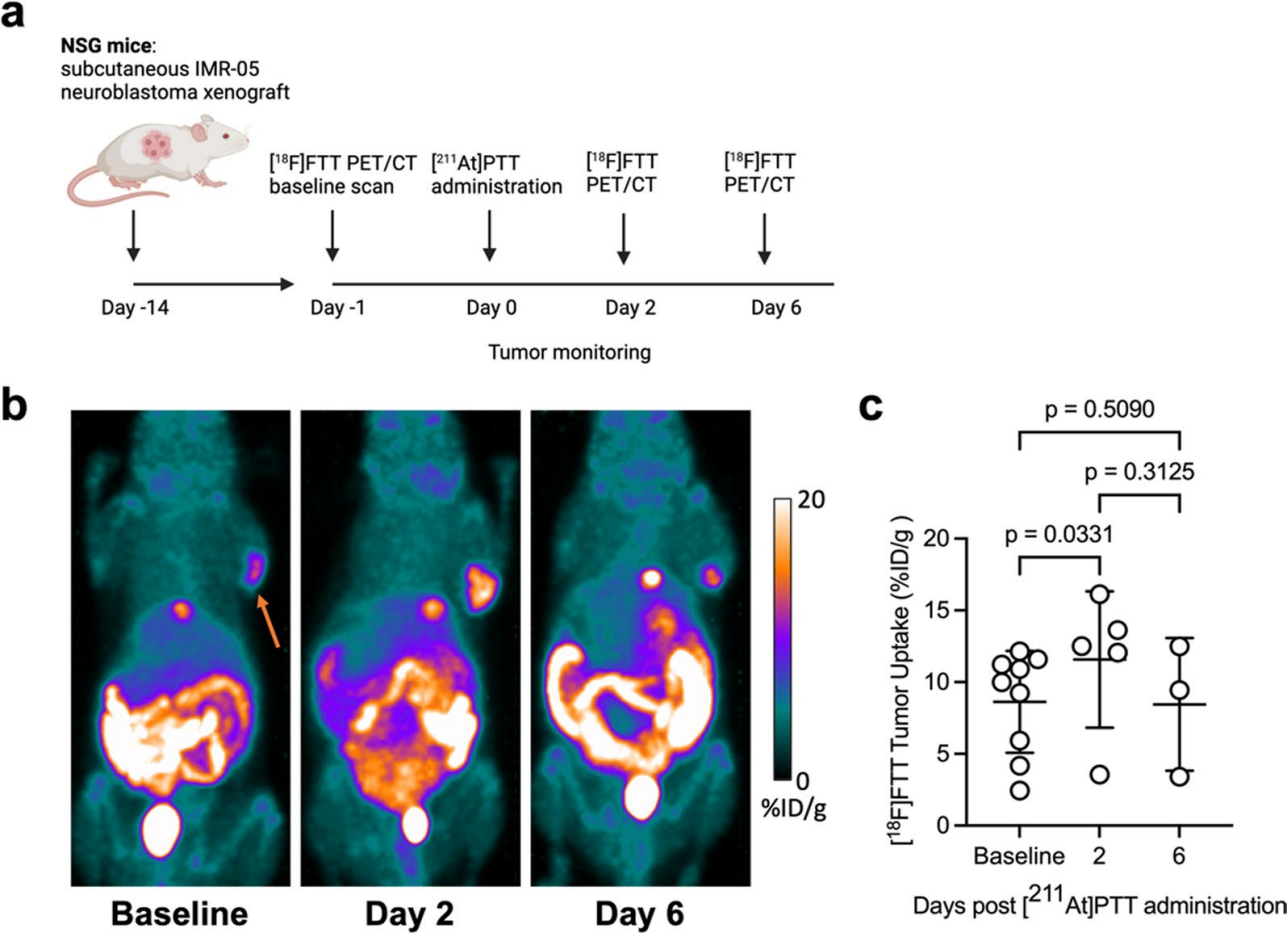
Preclinical imaging and therapy in IMR-05 xenograft mouse model. **a** Schematic of the preclinical study design evaluating the efficacy of a single 370 kBq dose of [^211^At]PTT with [^18^F]FTT PET/CT imaging. **b** Representative [^18^F]FTT PET/CT maximum-intensity projection images of tumor-bearing mice at baseline and post-[^211^At]PTT treatment, showing elevated tumor uptake (arrow) at day 2 compared to baseline. **c** Quantification of [^18^F]FTT tumor uptake over time, demonstrating a significant increase from baseline to day 2 (2-way ANOVA with Tukey–Kramer multiple comparisons test), followed by a return to baseline levels by day 6. Data are presented as mean ± SD (n = 3–9 per group)

We investigated whether [^211^At]PTT therapy induces transient upregulation of PARP1 expression in neuroblastoma xenografts and whether this effect could be quantitatively visualized using [^18^F]FTT PET/CT imaging. IMR-05 tumor-bearing mice (n = 9) were divided into a baseline group (n = 3, sacrificed after initial imaging) and a treatment group (n = 6) receiving [^211^At]PTT. Baseline measurements established pre-therapy PARP1 expression and tumor volume, while treated mice underwent longitudinal imaging and analysis. The study design for the [^18^F]FTT/[^211^At]PTT in vivo experiment is depicted in Fig. 1a. All 9 mice underwent [^18^F]FTT PET/CT imaging at baseline, 5 mice were imaged on day 2 (the PET images from 1 mouse were not evaluable and it was excluded), and 3 mice were imaged on day 6. PET/CT imaging with [^18^F]FTT with representative baseline and post-treatment images following [^211^At]PTT, are shown in Fig. 1b. Tumor uptake of [^18^F]FTT was quantified for each animal, and the percent change in tumor volume was also assessed. As shown in Fig. 1c, [^18^F]FTT tumor uptake was 8.6 ± 3.5%ID/g at baseline, 11.6 ± 4.8%ID/g on day 2, and 8.5 ± 4.6%ID/g on day 6. Pairwise comparisons indicated that [^18^F]FTT tumor uptake increased by 34.1% from baseline to day 2 (mean difference: 3.0%ID/g, *p* = 0.03), followed by a return towards baseline levels by day 6 (mean difference between baseline and day 6: -0.2%ID/g, *p* = 0.51).

**Fig. 2:**
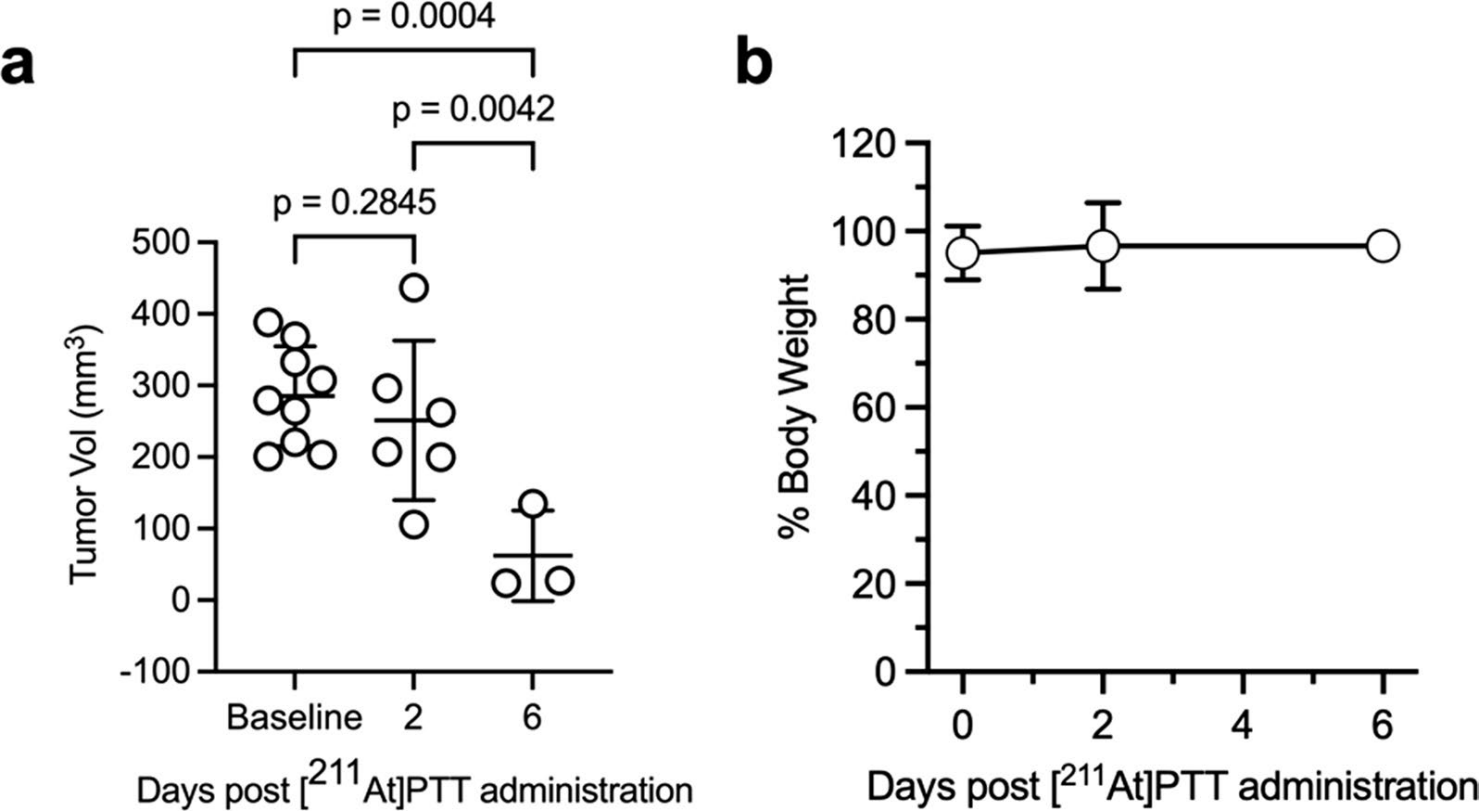
In vivo effect of single dose of [^211^At]PTT in IMR-05 xenograft mouse model. **a** Tumor volume measurements over time show significant reductions by day 6, as analyzed by 2-way ANOVA with Tukey–Kramer multiple comparisons test. **b** Mouse body weight remained stable across all time points. Data are presented as mean ± SD (n = 3–6 per group)

Analysis of tumor size over time showed that mean tumor volumes decreased by 11.8% from baseline to day 2, and by 77.2% from baseline to day 6 post-treatment, which represented a significant response to therapy (Fig. 2a). Overall, treated mice across all groups maintained a healthy weight throughout the study, defined as 100 ± 20% of their initial body weight (Fig. 2b). The [^211^At]PTT treatment and serial [^18^F]FTT PET/CT imaging were well-tolerated, with no observable adverse effects.

**Fig. 3:**
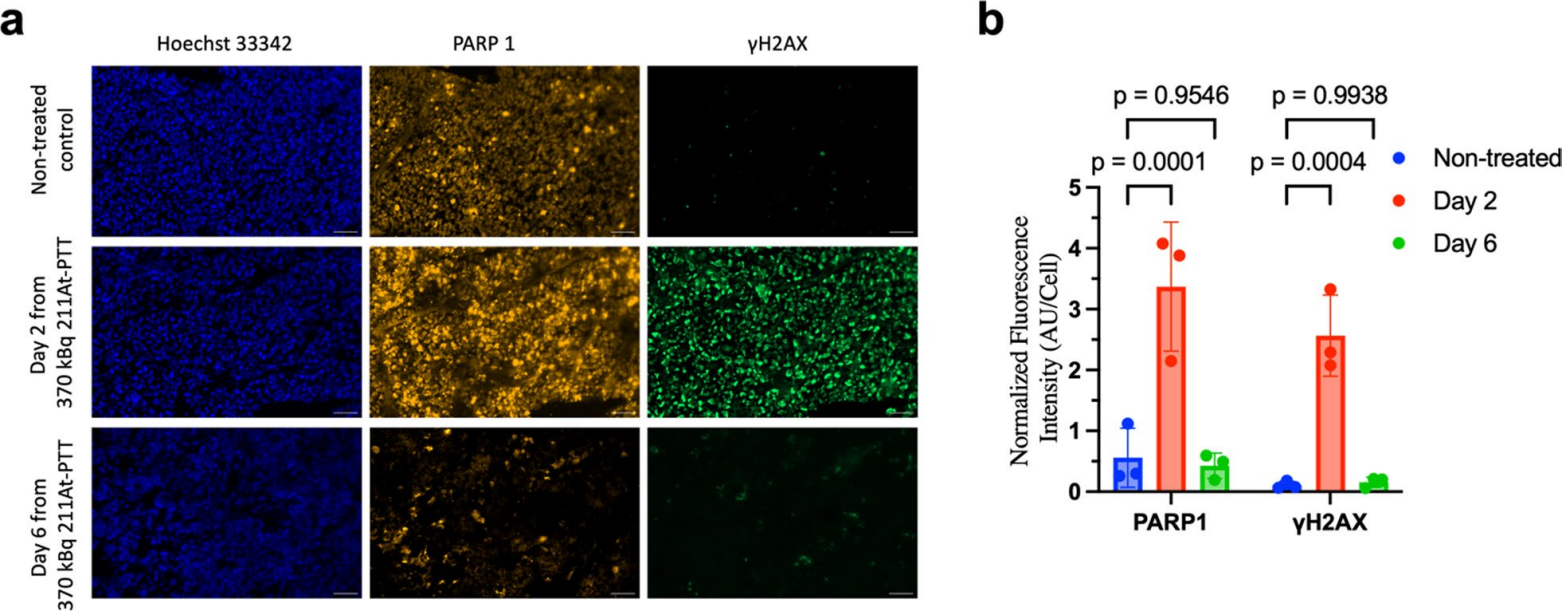
Immunofluorescence analysis of PARP-1-mediated DNA damage induced by [^211^At]PTT. **a** Representative images of tumor sections from mice treated with [^211^At]PTT, resected at the indicated time points. Scale is 50 µm. **b** Quantification of PARP1 and γH2AX expression in tumor sections from treated and non-treated mice. Data are presented as mean ± SD. Significant differences in PARP1 (*p* = 0.0001) and γH2AX expression (*p* = 0.0004) were observed between treated and non-treated groups on day 2 (2-way ANOVA with Tukey–Kramer multiple comparisons test)

Immunofluorescence analysis of tumors harvested from three mice at each time point (baseline, day 2, and day 6) confirmed comparable levels of nuclear PARP1 expression at baseline and day 6, with minimal γH2AX staining observed at baseline. Specifically, at day 2, a significant increase in γH2AX staining was detected (*p* = 0.0004), along with overlapping increase in PARP1 expression (*p* = 0.0001), indicating activation of the DNA damage response (Fig. 3a and b). This transient upregulation of PARP1 expression and DNA damage response was consistent with the increased [^18^F]FTT uptake observed on day 2, with both PARP1 expression and [^18^F]FTT uptake returning to baseline levels by day 6, further supporting the strong correlation between imaging findings and molecular markers of PARP1 activity (Pearson’s *r* = 0.73, *p* = 0.02) (Supplementary Fig. S3).

**Fig. 4:**
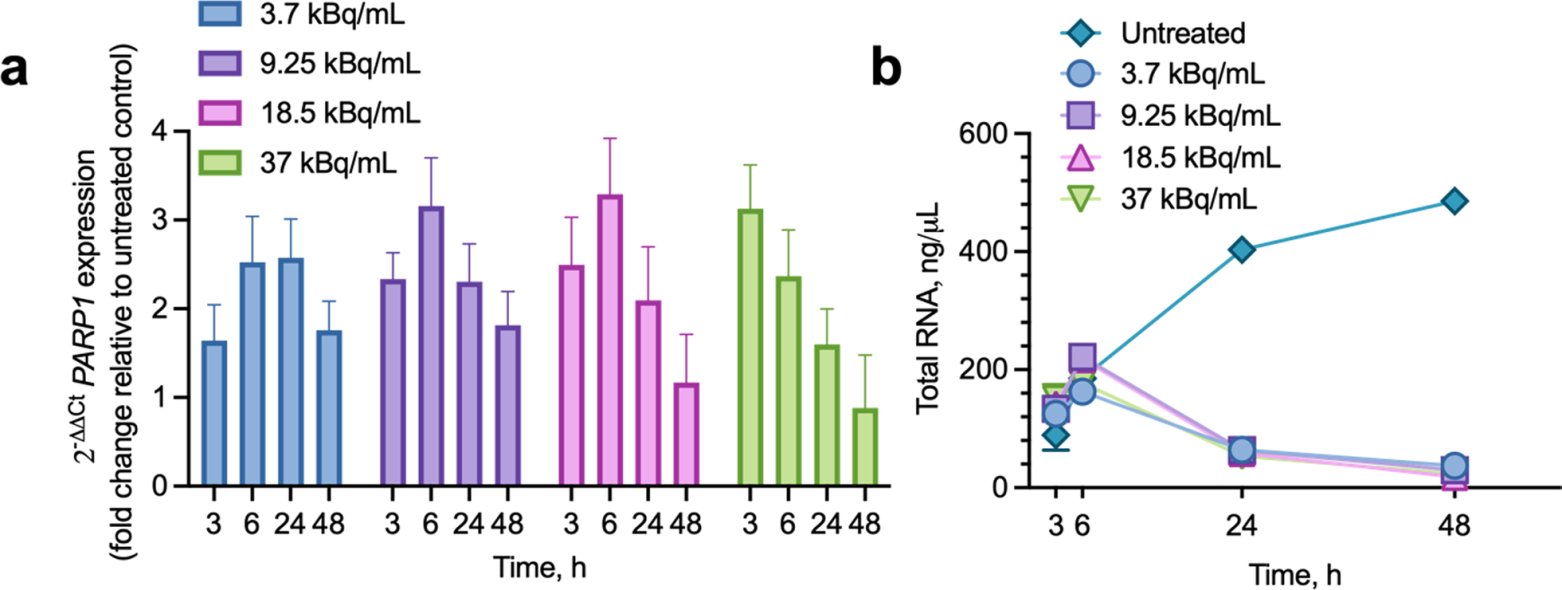
PARP1 expression and total RNA yields in IMR-05 cells treated with [^211^At]PTT **a** Relative *PARP1* gene expression (2^−ΔΔCt^ values, fold change relative to untreated control, normalized to B2M) across doses (3.7, 9.25, 18.5, and 37 kBq/mL) at 3, 6, 24, and 48 h. **b** Total RNA yields over the same time points and doses, compared to untreated controls, exhibiting an early peak at 6 h followed by a sharp decline by 24 and 48 h. Data represent the mean ± standard error of four replicates (n = 4) per condition.

In IMR-05 cells treated with [^211^At]PTT, PARP1 expression responded differently as doses increased. At the lowest dose (3.7 kBq/mL), PARP1 expression began to upregulate, peaking at 6–24 h post-treatment before declining by 48 h, while at intermediate doses (9.25, and 18.5 kBq/mL) PARP1 expression peaked at 6 h, and at the highest dose (37 kBq/mL) expression peaked at 3 h. At 3.7 kBq/mL, expression increased relative to untreated controls by 1.64-fold (± 0.80) at 3 h, 2.53-fold (± 1.03) at 6 h, and 1.76-fold (± 0.65) by 48 h. At 9.25 kBq/mL, expression increased by 2.33-fold (± 0.60) at 3 h, 3.16-fold (± 1.08) at 6 h, and 1.81-fold (± 0.76) by 48 h. At 18.5 kBq/mL, expression increased by 2.50-fold (± 1.07) at 3 h, 3.29-fold (± 1.26) at 6 h, and 1.17-fold (± 1.09) by 48 h. In contrast, the highest dose (37 kBq/mL) induced an early peak at 3 h with an increase of 3.13-fold (± 0.99), followed by an increase of 2.37-fold (± 1.05) at 6 h and 0.88-fold (± 1.20) by 48 h, reflecting greater cytotoxicity (Fig. 4a). This balance of PARP1 overexpression, with lower doses eliciting a delayed peak and the higher dose driving an earlier peak, highlights the potential for fractionation to optimize therapeutic outcomes.

Similarly to PARP1 expression, total RNA yields in IMR-05 cells treated with [^211^At]PTT varied over time (Fig. 4b). In untreated controls, RNA yields rose steadily from 3 to 48 h due to cell proliferation. In treated cells across all doses (3.7–37 kBq/mL), RNA yields increased peaking at 6 h, before declining by 24 and 48 h, indicative of progressive cell death. These data suggest that timing and dose fractionation could modulate PARP1 expression to enhance efficacy while minimizing toxicity, though further studies are needed to determine the optimal regimen for fractionated dose administration.

## Discussion

This study demonstrates that [^211^At]PTT, a PARP1-targeted alpha-emitting radiopharmaceutical, enhances DNA damage and transiently upregulates PARP1 expression in neuroblastoma xenografts. This approach has the potential to address the challenges of relapse and resistance associated with conventional therapies [12, 13]. The observed increase in γH2AX staining and PARP1 expression at day 2 post-treatment, detected via [^1^^8^F]FTT PET/CT and immunofluorescence, supports the hypothesis that sublethal alpha damage increases tumor vulnerability. Sublethal damage here denotes doses insufficient to eradicate all cells in a single administration, leaving residual cells reliant on PARP1 for survival. This dependency creates a therapeutic window for subsequent interventions, as PARP1 hyperactivity sensitizes cells to further treatment with PARP1-targeted therapies. This transient PARP1 upregulation suggests that fractionated therapy could exploit this vulnerability, potentially enhancing therapeutic efficacy. The dynamic changes in [^1^^8^F]FTT uptake, with a significant increase at day 2 followed by a return to baseline by day 6, reflect the temporal modulation of PARP1 activity. This decline may indicate reduced DNA repair capacity or cell loss, underscoring the importance of timed dosing. Previous studies have shown that fractionated alpha therapy can enhance efficacy while minimizing toxicity [4, 14, 15], and our results suggest this approach could be particularly beneficial in neuroblastoma, where high PARP1 activity correlates with aggressive tumor behavior [16, 17]. The current study utilized a small sample size, which was sufficient to detect significant differences in [^18^F]FTT uptake and tumor volume, but larger cohorts are needed to confirm these findings. Additionally, the baseline group provided only initial measurements, precluding direct comparison of tumor progression in untreated vs. treated animals. Future work will incorporate longitudinal untreated controls and sham-treated groups to isolate treatment effects. In vitro, PARP1 expression peaked 3–24 h post-[^211^At]PTT treatment, depending on the dose, consistent with its activation to repair DNA damage, while its relative decline at higher doses may reflect a shift toward cell death pathways [18]. Although PARP1 expression was increased 48 h post-[^211^At]PTT treatment both in vivo and at lower doses in vitro, the in vitro data suggest that PARP1 expression may peak earlier. However, directly comparing the in vivo and in vitro results is difficult due to differences in drug exposure and biological complexity. In vitro, direct [^211^At]PTT delivery induces rapid PARP1 mRNA upregulation, while in vivo, delayed tracer uptake, heterogeneous tumor penetration, clearance of tracer, and post-transcriptional regulation likely shift the protein-level peak to a later time point. Critically, [^18^F]FTT PET captures this functional PARP1 activity, which aligns with γH2AX-confirmed DNA damage. The transient surge represents a therapeutically exploitable window, where fractionated dosing could amplify cytotoxicity. A hypothetical fractionated regimen administering repeat doses every 24–48 h could exploit repair hyperactivity while minimizing off-target effects. Future studies incorporating earlier time points and PET-guided adaptive dosing will refine this paradigm. This robust PARP1 response highlights the therapeutic potential of [^211^At]PTT and warrants further investigation into PARP1-targeted strategies.

## Conclusions

Our findings identified transient PARP1 upregulation post-[^211^At]PTT therapy which could be exploited in future studies using fractionated treatment regimens to enhance tumor control and minimize toxicity. [^18^F]FTT PET/CT imaging provides a valuable tool for monitoring PARP1 activity, enabling precise optimization of therapy for high-risk neuroblastoma.

## Supplementary Information

Additional file1.

## Data Availability

All data generated or analyzed during this study are included in this published article and its supplementary information files.

## Abbreviations

PARP1: Poly [ADP-ribose] polymerase 1
[^211^At]PTT: [^211^Astatine]-parthanatine
[^18^F]FTT: [^18^Fluorine]-fluorthanatrace
PET/CT: Positron Emission Tomography/Computed Tomography
γH2AX: Gamma H2A histone family member X
OCT: Optimal cutting temperature
NSG: Non-obese scid gamma
