# Supplementary material for: PARP1-targeted alpha therapy enhances target expression
Source: EJNMMI Res. 2025 Jun 1;15:63. doi: 10.1186/s13550-025-01256-0 (PMC12127250; doi:10.1186/s13550-025-01256-0)
Supplement: Supplementary file 1 — Additional file1. [file 13550_2025_1256_MOESM1_ESM.docx]

**Supplementary information**

**PARP1-targeted alpha therapy enhances target expression**

Hasan Babazada^1^, Paul Martorano^1^, Hsiaoju Lee^1^, Shuyao Geng^1^, Vandana Batra^2^, John M Maris^2^, Daniel A Pryma^1,2^, Sarah B Gitto^1^, Michael D Farwell^1^

^1^Perelman School of Medicine at the University of Pennsylvania, Philadelphia, PA, USA.

^2^Children's Hospital of Philadelphia, Philadelphia, PA, USA.

^3^Abramson Cancer Center at the University of Pennsylvania, Philadelphia, PA, USA.

*Corresponding author

[hasan.babazada@pennmedicine.upenn.edu](mailto:hasan.babazada@pennmedicine.upenn.edu)

3620 Hamilton Walk, John Morgan Bldg., Philadelphia, PA 19104, USA


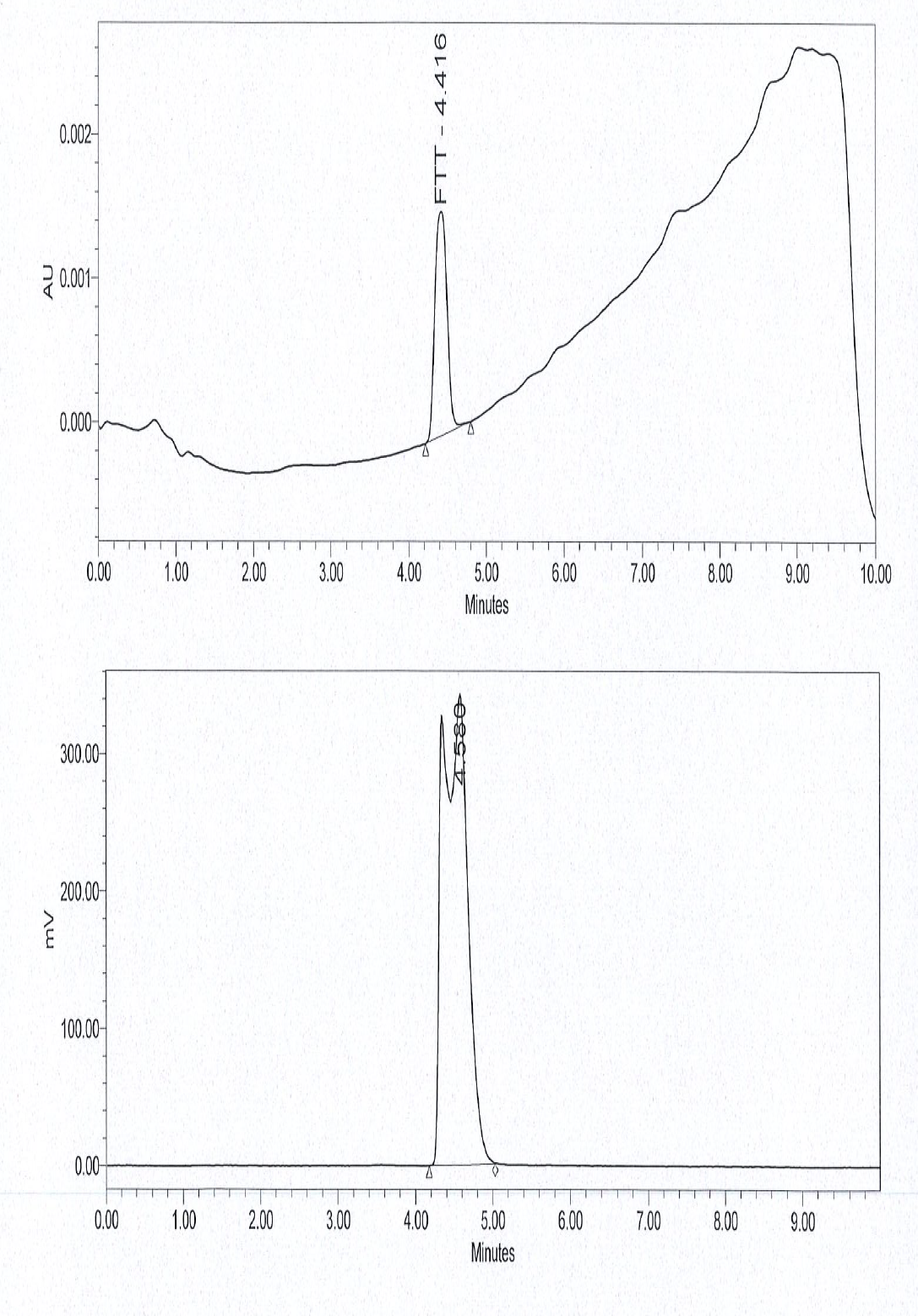


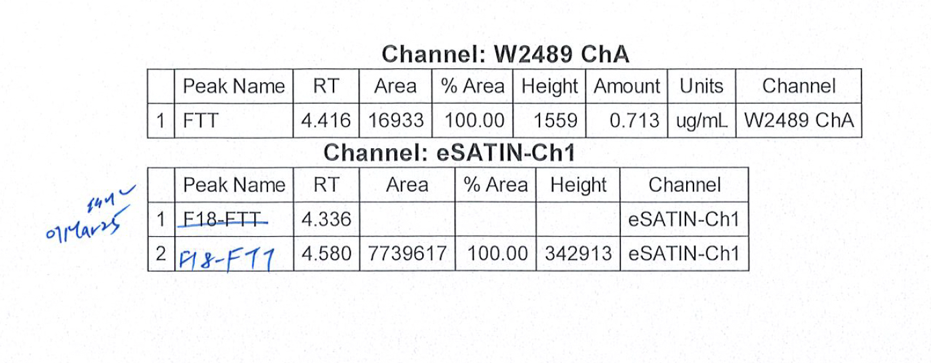


**Fig. s1** Representative chromatogram demonstrating the radiochemical purity of [^18^F]FTT. The main peak at a retention time of 4.5 minutes corresponds to [^18^F]FTT.


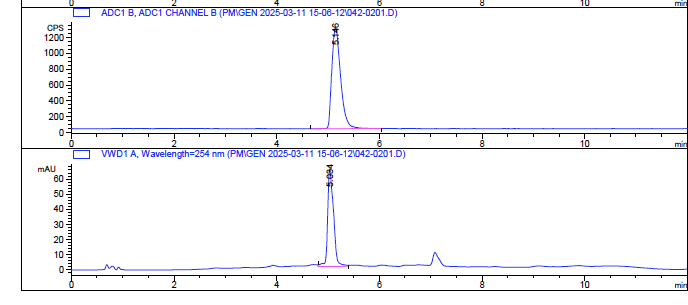


**Fig. s2** Representative chromatogram demonstrating the radiochemical purity of [^211^At]PTT. The main peak at a retention time of 5.146 minutes corresponds to [^211^At]PTT.

**Fig. s3** **Correlation between PARP1 expression and [^18^F]FTT tumor uptake.** Each point represents an individual paired measurement. The solid line indicates the linear least-squares regression fit to the data. The area between the dashed lines denotes the 95% confidence interval. Pearson’s correlation analysis yielded a correlation coefficient of *r*=0.73 and p=0.02, demonstrating a strong and statistically significant positive linear relationship between PARP1 expression and [^18^F]FTT tumor uptake.
